# A practical guide to managing hypertension, hyperlipidemia, and hyperglycemia in patients with chronic myeloid leukemia

**DOI:** 10.3389/fmed.2022.1025392

**Published:** 2022-12-07

**Authors:** Khalid Ahmed, Rasha Kaddoura, Mohamed A. Yassin

**Affiliations:** ^1^Department of Hematology, National Center for Cancer Care and Research (NCCCR), Hamad Medical Corporation (HMC), Doha, Qatar; ^2^Pharmacy Department, Heart Hospital, Hamad Medical Corporation (HMC), Doha, Qatar

**Keywords:** chronic myeloid leukemia, hypertension, hyperglycemia, hyperlipidemia, dasatinib, nilotinib, ponatinib, tyrosine kinase

## Abstract

Tyrosine kinase inhibitors (TKIs) have significantly improved the prognosis of chronic myeloid leukemia (CML) since their approval. Although safe in general, TKIs carry concerns about cardiovascular adverse events. Hypertension, diabetes, and dyslipidemia are among the most common baseline comorbidities among CML patients. Guidelines for the management of the existing comorbidities or those related to TKI therapy are lacking. This paper will review hypertension, hyperglycemia and hyperlipidemia reported in CML patients or associated with TKI therapy and then propose a simple guide on their management.

## Introduction

Chronic myeloid leukemia (CML) is a myeloproliferative neoplasm caused by a fusion gene that encodes for the oncoprotein BCR-ABL, with constitutive tyrosine kinase activity (1); the target for tyrosine kinase inhibitor(s) [TKI(s)] (2). The introduction of the first generation TKI (imatinib) in 2001, substantially improved CML prognosis with reported survival figures comparable to that of the general population (1, 3). The striking results continued with the availability of the second (bosutinib, dasatinib, nilotinib) and third (ponatinib) generation TKIs, by obtaining faster and more effective deep molecular responses, but at the expense of the toxicity profile (1). TKIs are generally safe, and most patients experience mild and transient adverse events (3). However, there are major concerns about the reported cardiovascular adverse events and toxicity in randomized and observational trials (1). CML patients usually have baseline comorbidities or experience cardiovascular events that precluded them from enrolling in the CML landmark trials (4, 5). The evidence for the medical management of the existent or new comorbidities is scarce (3), and dedicated guidelines are lacking. Herein, this review will discuss some of the most common comorbidities reported in CML patients or associated with their TKI therapy and draft a practical guide on their management in the daily clinical practice.

## Baseline comorbidities and on-TKI incidence of cardiovascular events

Patients with CML usually have baseline comorbidities or develop new morbidities when starting TKI therapy. An analysis of data from the United States (*n* = 1,639) reported an incidence of cardiovascular diseases (e.g., myocardial infarction, angina, stroke, peripheral arterial disease, atherosclerosis, and heart failure) of 33% and cardiovascular risk factors (e.g., diabetes mellitus, hypertension, smoking and high blood cholesterol) of 77.7% in CML patients at five-year follow-up. At 1 year follow up, the age and gender standardized annual rates of cardiovascular conditions for CML patients were significantly higher by a factor of 1.3 to 3.5 compared to the general population. Similarly, the standardized annual rates for hypertension, diabetes, and obesity were higher by 20–40% (*p* < 0.001) (6). Another retrospective analysis from the MarketScan Commercial and Medicare databases (*n* = 2,296) demonstrated that 41% of those who newly started TKI therapy had at least one comorbid disease. The most prevalent comorbidity among patients with CML was heart disease. Its prevalence (45% among Medicare-insured and 23% among commercially insured individuals) was higher than that among the United States population which ranged from 12% (age 47–64) to 37% (age ≥75). Diabetes comes second with a prevalence of 24% among Medicare-insured and 16% among commercially insured CML patients that is also higher than that of the general population (13% for individual aged 45–64 and 20% for those aged ≥75 years) (7). Elderly patients are more likely to have multiple comorbidities; 56% of patients with a median age of 78.5 years at the time of diagnosis had two or three comorbidities (8). Interestingly, although the presence of comorbidities did not affect the success of CML treatment, it has negatively impacted the overall survival. This was demonstrated in the analysis of the CML Study IV (*n* = 1,519) that examined the impact of comorbidities, using the Charlson Comorbidity Index (CCI), at the CML diagnosis on remission rate and overall survival. There was a significant negative association between comorbidities and overall survival. The CCI of 2, 3–4, 5–6, and ≥7 was associated with 93.6, 89.4, 77.6, and 46.4% probabilities of eight-year overall survival, respectively. In a multivariate analysis, CCI was the strongest predictor of the overall survival. The benefit from imatinib therapy was significantly higher in those with multiple comorbidities. However, comorbidities had no influence on remission rates or disease progression. Thus, comorbidities may be a determinant for survival as opposed to the CML disease itself (9).

A study which investigated the incidence of cardiovascular and arteriothrombotic events in CML patients treated with TKIs (*n* = 531), found that 45 and 9% of patients developed cardiovascular and arteriothrombotic adverse events, respectively. The most common morbidity was hypertension (33%) (10). With regard TKI therapy, the second generation TKIs (at least nilotinib and dasatinib) have 1.5–2 times higher cardiovascular risk than with imatinib. The incidence of myocardial infarction in patients treated with dasatinib or nilotinib increased by 2.4 or 3.6 times in comparison with imatinib (3). Whereas ponatinib, the most potent and third generation TKI, had the highest incidence ratios for cardiovascular [40.7; 95% confidence interval (CI): 27.9–59.4] and arteriothrombotic (9.0; 95% CI: 4.1–20.1) adverse events, moreover, treatment with ponatinib was associated with significantly higher incidence rate ratios for cardiovascular (4.62; 95% CI: 2.7–7.7; *p* < 0.0001) and arteriothrombotic (6.38; 95% CI: 1.8–21.8; *p* < 0.0001) adverse events compared with imatinib when using a multivariable analysis. Overall, newer TKIs, especially ponatinib, may carry higher risk for cardiovascular adverse events (10). Given the lack of head-to-head comparison between various TKIs and inconsistency in the reporting of adverse events, precise quantitation of this increased risk is not possible. For the same reason, using data from the randomized trial that compared bosutinib, dasatinib, or nilotinib with imatinib may allow an indirect comparison between the second-generation drugs (3). The cardiovascular toxicities of bosutinib are considered rare. In the BELA (Bosutinib Efficacy and Safety in Newly Diagnosed CML) trial, cardiotoxicities (including heart failure) were similar between bosutinib and imatinib arms (11). A meta-analysis of 10 randomized trials did not show statistically significant difference in the risk of vascular occlusive complications between bosutinib and imatinib (12).

### Mechanism of cardiovascular morbidities or adverse events

The mechanisms of the TKIs adverse effects are related to inhibiting the main BCR-ABL1 tyrosine kinase (i.e., on-target effects) and other kinases that are not involved to the CML pathogenesis (off-target effects) (1). The activity and potency of the approved TKIs against non-BCR-ABL kinases varies and targets vascular endothelial growth factor receptors (VEGFR) 1–3, angiopoietin receptor TIE-2, fibroblast growth factor receptors, and platelet-derived growth factor receptors A and B (2). The resultant off-target effects are believed to increase cardiovascular risk. The spectrum of cardiovascular adverse effects of each TKI also vary and can be influenced by patient's age, gender, comorbidities, and the existence of other cardiovascular risk factors such as smoking, diabetes, dyslipidemia, obesity, drug-drug interaction and so forth. The mechanism of cardiovascular adverse effects caused by TKIs is not fully explained but it might be multifactorial. Different factors may lead to endothelial damage and atherosclerosis, hypertensive effect, metabolic impairment, and mast-cell disruption (1). TKIs can also affect the platelets activities such inhibiting their activation, spreading, and aggregation (2). Table 1 summarizes cardiovascular adverse effects of TKIs and their potential mechanisms (1–3).

**Table 1:** Mechanism of cardiovascular adverse events of TKIs.

**Generation**	**TKI**	**Cardiovascular effect**	**Mechanism**	**Preferred in**	**Less preferred in**
First	Imatinib	• Potential metabolic and cardiovascular functional benefit	• Improves metabolic profile of DM patients • Does not affect CV risk profile • Does not impair vascular endothelial and microvascular endothelial cell proliferation and survival • Inhibits mast-cell KIT receptor but does not impair endothelial cell regeneration or sensitize the endothelium to atherosclerosis generation^#^	CVD, DM, PAD, PAH	-
Second	Bosutinib	• No potential CV adverse events	-	CVD, DM, PAD, PAH	-
	Dasatinib	• PAH	• Possible hypoxic pulmonary vasoconstriction responses reduction • Induces human umbilical venous endothelial cells apoptosis and necrosis (*in vitro*)	DM, (PAD?)	CVD, PAH
	Nilotinib	• Arterial hypertension • Ischemic events • Hyperglycemia • Worsening pre-existing DM	• Inhibits vascular endothelial and microvascular endothelial cell proliferation and survival • Increases expression of ICAM-1, VCAM-1, and E-selectin as pro-atherogenic surface molecules in endothelial cells (*in vitro*) • Inhibits post-insulin receptor pathway and decreases insulin sensitivity • Potently inhibits VEGFR leading to HTN^*^ • Inhibits mast-cell KIT receptor, thus impairs endothelial cell regeneration, or sensitizes the endothelium to atherosclerosis generation^#^	PAH	CVD, DM, PAD
Third	Ponatinib	• Peripheral arterial obstructive disease • Ischemic events	• Inhibits vascular endothelial and microvascular endothelial cell proliferation and survival • Inhibits VEGFR2 (KDR) leading to proteinuria and thrombotic microangiopathy • Inhibits angiopoietin receptor TIE-2 (KDR) and FGFR kinases leading to vascular toxicity • Induces human umbilical venous endothelial cells apoptosis and necrosis (*in vitro*) • Potently inhibits VEGFR leading to HTN^*^ • Inhibits mast-cell KIT receptor, thus impairs endothelial cell regeneration, or sensitizes the endothelium to atherosclerosis generation^#^	-	PAH

CVD, cardiovascular disease; DM, diabetes mellitus; PAD, peripheral arterial disease; PAH, pulmonary arterial hypertension; TKI, tyrosine kinase inhibitor; VEGF/VEGFR, vascular endothelial growth factor/receptors.

*VEGF physiologically supports nitric oxide (NO)-dependent arterial vasodilation and upregulates NO production acting on the endothelial cells, thus regulating the basal arterial tone and pressure levels. VEGF and VEGFR inhibitors determine the blocking of this mechanism and may cause hypertension.

^#^Mast cells have an important role in vascular tissue repair, producing and releasing heparin and other bioactive tissue plasminogen activator.

The (?) symbol indicates some evidence but not strong evidence for its preference in PAD.

### Hypertension

The International Society of Hypertension/American Heart Association's definition of hypertension is shown in Table 2 (13). The available evidence showed that TKIs are associated with an increased hypertension incidence of varying degrees, specifically with ponatinib which is approved for the treatment of resistant CML cases particularly those carrying the T315I mutation. This can be explained by the potent inhibitory effect of ponatinib on VEGFR-2 leading to a decrease in nitric oxide production (i.e., a potent vasodilator) and an increase in endothelin production (2). Hypertension was the most frequent adverse event (33%) among 531 patients treated with four TKIs, imatinib (in two doses, 400 and 800 mg), nilotinib, dasatinib, and ponatinib. New-onset or worsening hypertension rates were observed in 15% and 18% of patients. The readings of blood pressure measurements, both systolic and diastolic, increased significantly after starting therapy for all TKIs. The change in blood pressure measurement before and after therapy did not differ among the studied TKIs (10). The incidence of hypertension with ponatinib can reach up to 68% of patients (14). A dose dependent pattern of increase in cardiovascular risk was also noted in ponatinib trials especially in older patients with history of diabetes or previous ischemic events (2). Dasatinib was associated with an increase in pulmonary hypertension by 5% at 5 years of follow-up (15).

**Table 2:** Definition of hypertension.

**Category**	**Systolic BP (mmHg)**		**Diastolic BP (mmHg)**
Normal	<130	and	<85
High normal BP	130–139	and/or	85–89
Garde 1	140–159	and/or	90–99
Grade 2	≥160	and/or	≥100

### Hyperlipidemia

There are variable classifications of hyperlipidemia, an example is shown in Table 3 (16). The incidence of hyperlipidemia in CML patients on dasatinib or nilotinib as first or second line therapy was reported as 46.4 per 1,000 Patient-year (95% CI: 33.00–63.45) for dasatinib and 74.6 per 1,000 Patient-year (95% CI: 50.70–105.94) for nilotinib. The latter had higher rate of incidence (hazard ratio 1.75; 95% CI: 1.07–2.87) (17). A study evaluating plasma lipid profile and cardiovascular risk in a series of 27 CML patients at 1 year of follow-up found that nilotinib significantly increased total, low- and high-density lipoprotein cholesterol (TC, LDL-C and HDL-C) after 3 months of therapy initiation (18). As a result, the percentage of patients with less-than-optimal LDL-C moved up from 48.1 to 88.9% at 12 months of therapy with 22.2% of the patients needed to start cholesterol lowering treatment. This was reflected in an 11% increase in global cardiovascular risk. Thus, due to the known atherogenic potential of LDL-C, the authors suggested frequent monitoring of lipid profile along with life-style modifications and/or drug therapy if indicated once initiating treatment with nilotinib. In a recent retrospective study, which evaluated the long-term effect of imatinib on the glycemic and lipid profiles of CML patients, significant reductions in LDL-C [17.8 mg/dL, interquartile range (IQR) −1.3–34.0; *p* < 0.001] and triglycerides (25.0 mg/dL, IQR −2.3–58.3; *p* < 0.001) levels were recorded at 12 months of treatment independent of patient demographics or usage of statin therapy (19).

**Table 3:** Definitions of hyperlipidemia.

**Category**	**Level mg/dl (mmol/l)**
***Triglycerides***	
Moderate hypertriglyceridemia	150–499 (1.7–5.6)
Moderate to severe hypertriglyceridemia	500–999 (5.65–11.3)
Severe hypertriglyceridemia	≥1,000 (>11.3)
***Total cholesterol***	
Desirable TC	<200 (5.2)
Borderline high TC	200–239 (5.2–6.1)
High TC	>239 (6.1)
***LDL-C***		
Optimal LDL-C	<100 (2.6)
Near optimal LDL-C	100 to (2.6–3.3)
Borderline high LDL-C	130–159 (3.4–4.1)
High LDL-C	160–189 (4.1–4.9)
Very high LDL-C	>189 (4.9)
***HDL-C***	
Low HDL-C (Men)	<40 (1.03)
Low HDL-C (women)	<50 (1.30)

HDL, high-density lipoprotein cholesterol; LDL-C, low-density lipoprotein cholesterol; TC, total cholesterol.

### Hyperglycemia

The criteria for the diagnosis of hyperglycemic conditions as endorsed by the American Diabetes Association are presented in Table 4 (20). A study investigated the effect of the first and second TKI generations on glucose metabolism in CML patients without previous hyperglycemic disorder, found that diabetes mellitus or impaired fasting glucose were identified in 25% of imatinib- and dasatinib-treated patients, and in 33% in nilotinib cohort (*p* = 0.39 vs. imatinib and *p* = 0.69 vs. dasatinib). Metabolic syndrome was seen in 42.4% of imatinib-treated, 37.5% of dasatinib-treated, and 36.1% of nilotinib-treated patients (*p* = 0.46 vs. imatinib and *p* = 0.34 vs. dasatinib). The authors concluded that although nilotinib does not seem to induce hyperglycemic disorders to a significantly higher extent than dasatinib or imatinib, it has a worse glucometabolic profile (21). At a long-term follow-up of a phase III trial on the efficacy and safety of nilotinib vs. imatinib in newly diagnosed CML patients in chronic phase, hyperglycemia was reported in 50% of patients on nilotinib 300 mg twice daily, 53% of those on nilotinib 400 mg twice daily compared with 31% of patients treated with imatinib 400 mg per day (22). Despite the previously mentioned rates, there is no real-life data that accurately estimates the incidence of diabetes, impaired fasting glucose, or metabolic syndrome in CML patients on TKI therapy. In a recent retrospective study, which evaluated the long-term effect of initiating imatinib on glycemic and lipid profiles in CML patients (*n* = 611), significant reductions in HbA1c (0.53%, IQR 0.09–1.19; *p* < 0.001) and fasting blood glucose (10.2 mg/dL, IQR −3.5–32.2; *p* < 0.001) were observed in patients with diabetes (*n* = 118) during the first year of therapy independent of anti-diabetic medications use or patients' demographics. The reductions persisted through the second year of treatment. The authors concluded that imatinib is associated with a sustained metabolic benefit (19).

**Table 4:** Diagnosis of hyperglycemia.

**Category**	**IFG FPG mg/dl (mmol/l)**	**HbA1c (%)**	IGT PG^*^mg/dl (mmol/l)	**Random PG mg/dl (mmol)**
Prediabetes	100–125 (5.6–6.9)	5.7–6.4	140–199 (7.8–11)	-
Diabetes mellitus	≥126 (7.0)	≥ 6.5	≥200 (11.1)	≥ 200 (11.1)^#^

FPG, fasting plasma glucose; HbA1c, glycated hemoglobin; IGT, impaired glucose tolerance; PG, plasma glucose.

* 2-h during 75-gram OGTT (oral glucose tolerance test).

# In patients with classic symptoms of hyperglycemia or hyperglycemic crisis.

## Guide to management of comorbidities

### Prevention

**Figure 1:**
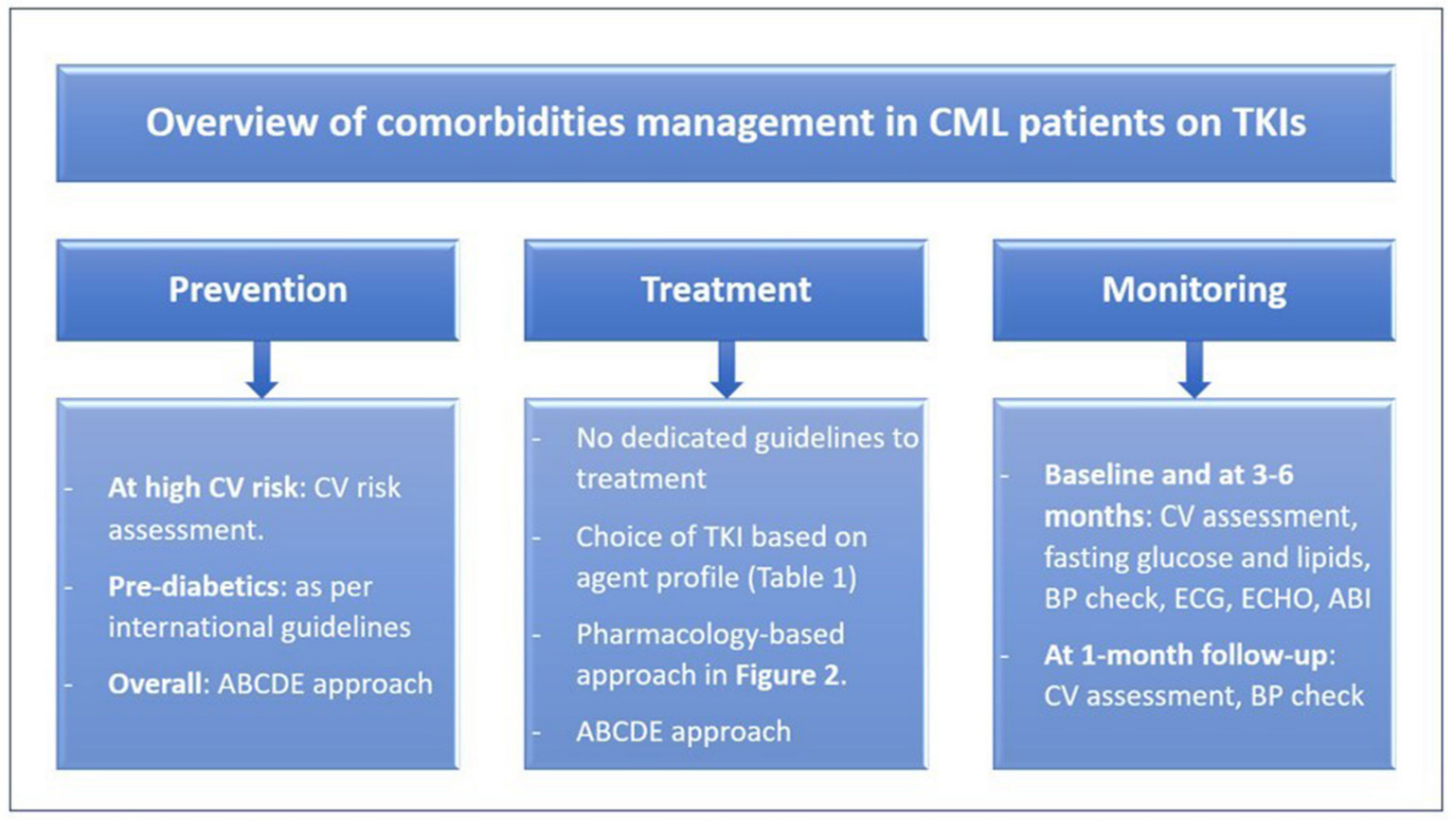
Overview of comorbidities management in CML patients on TKIs. ABCDE, assessment of risk, antiplatelet therapy, BP management, cholesterol management, cigarette/tobacco cessation, diet and weight management, diabetes prevention and treatment, and exercise; ABI, ankle-brachial index; BP, blood pressure; CML, chronic myeloid leukemia; CV, cardiovascular; ECG, electrocardiogram; ECHO, echocardiogram; TKIs, tyrosine kinase inhibitors.

Cardiovascular risk can be reduced through adopting lifestyle modifications and preventing risk factors occurrence (1, 23). Improving modifiable cardiovascular risk factors (e.g., hypertension, dyslipidemia, cigarette smoking, inactivity, obesity) after the diagnosis of CML is preferrable. The preventative strategy starts with baseline patient evaluation and risk assessment (e.g., SCORE charts, Framingham risk score), appropriate choice of TKI treatment, and proper therapy for primary and secondary prevention of cardiovascular diseases (1). The “ABCDE” approach has been recommended for the primary and secondary prevention of cardiovascular diseases in the general population (3, 23). It was proposed for CML patients treated with TKIs as well, especially those with comorbidities (2, 3). ABCDE refers to assessment of risk, antiplatelet therapy, blood pressure management, cholesterol management, cigarette/tobacco cessation, diet and weight management, diabetes prevention and treatment, and exercise (Figure 1) (23).

### Selection of TKI based on comorbidities

**Figure 2:**
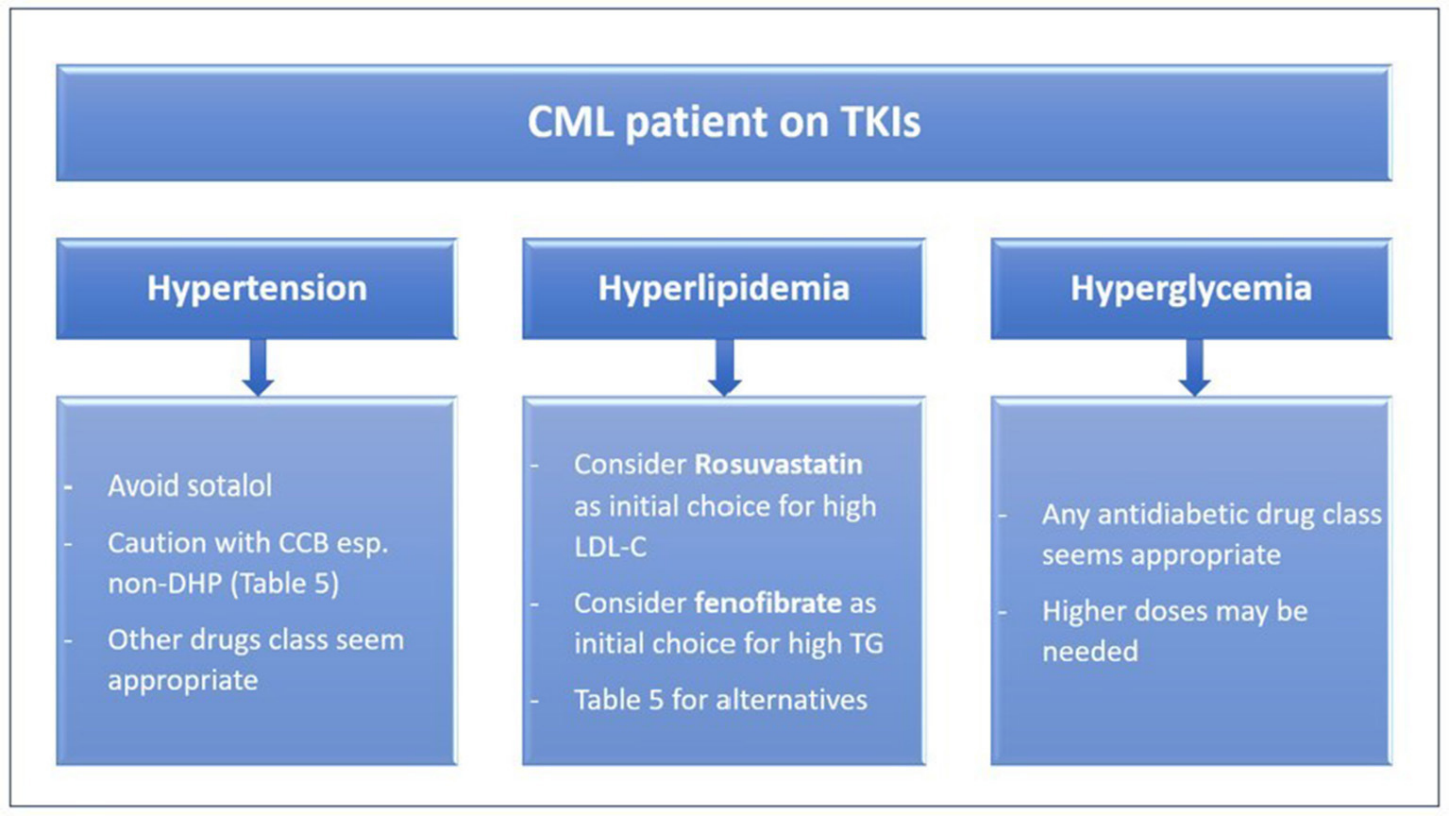
Pharmacology based approach to treating comorbidities in CML patients on TKIs. CCB, calcium channel blockers; CML, Chronic myeloid leukemia; LDL-C, low-density lipoprotein cholesterol; non-DHP, non-dihydropyridine, TG, triglycerides, TKIs, tyrosine kinase inhibitors.

The treatment of patients with existent or new comorbidity remains empiric. It should target two elements: selecting the most appropriate TKI (Table 1) and aggressive management of the comorbidities (3, 23) (Figure 2). The selection of a TKI depends on the expected adverse effect, comorbidities, disease characteristics, and patient preference (2). For instance, as strongly recommended by the 2020 European LeukemiaNet, ponatinib and nilotinib should be avoided in patients with arterial vascular disease (24), due to the associated high rate of vascular events with their use (2). Furthermore, nilotinib causes hyperlipidemia and hyperglycemia, thus it is not suitable for CML patients having such morbidities (3).

To reduce the dose-dependent cardiovascular adverse events, minimized doses of second and third generation TKIs can be considered to maintain the therapeutic benefit in those who attained adequate response after starting TKI therapy with full-dose regimen as suggested by observational studies (1, 2). A five-year analysis of the DASISION (Dasatinib vs. Imatinib Study in Treatment-Naïve Chronic Myeloid Leukemia Patients) trial demonstrated that reducing TKI dose for any reason did not reduce major molecular response (MMR) (i.e., efficacy was not affected) with no safety concern (25). In patients newly diagnosed with CML in chronic phase, dasatinib approved standard dose (i.e., 100 mg daily) can cause pleural effusion and myelosuppression. Naqvi et al. found that the use of a lower dose (i.e., 50 mg daily) is safe and effective in early therapy for CML in chronic phase (*n* = 60). Complete cytogenetic response was attained in 86% of patients at 6 months of therapy, with faster and higher rates than that with the standard dose (73%) or imatinib (59%). The MMR rate was 79% at 12 months. Overall, the therapy was well-tolerated, only one patient (1%) experienced pleural effusion, which necessitated dose reduction to 20 mg daily. Treatment interruption for 7–14 days was unavoidable in only 12% of the patients (26). Interruption of TKI therapy is possible and safe in certain cases (1). Another important factor to consider is the drug-drug interactions between TKIs and drugs prescribed to treat the comorbidities. For example, pravastatin and rosuvastatin are less interactive with TKIs than atorvastatin or simvastatin (3, 27) (Table 5).

**Table 5:** Drug-drug interactions between TKI and therapy of common conditions.

	**Imatinib, Nilotinib CYP3A4 Inhibitors (Moderate)**	**Bosutinib, Dasatinib, Ponatinib**
*Antidiabetic agents*		
All classes	For nilotinib **Moderate DDI**: monitor blood glucose more frequently; increase antidiabetic agent doses, or add additional agents Hyperglycemia-Associated Agents may diminish the therapeutic effect of Antidiabetic Agents **MOI**: opposite effects	-
Lipid lowering agent^*^		
Statin	Atorvastatin, lovastatin, simvastatin **Moderate DDI**: monitor for increased statin adverse effects (e.g., myopathy) CYP3A4 Inhibitors may increase serum concentration of statin **MOI**: inhibition of CYP3A4	-
Fibrates	Imatinib- Gemfibrozil **Moderate DDI**: monitor therapy Gemfibrozil may decrease serum concentration of imatinib **MOI**: possible inhibition of imatinib intestinal absorption and conversion to active metabolite (CYP1C8)	-
*Antihypertensive agents* ^$^		
Beta-blockers	-	Dasatinib-Sotalol (QT-prolonging agent; highest risk) **Severe DDI**: consider alternatives or monitor for QT-prolongation and its risk factors Sotalol may enhance QTc-prolonging effect of Dasatinib **MOI**: additive effect
DHP-CCB	Amlodipine (minor), felodipine (moderate), nifedipine (moderate) **DDI**: monitor for increased DHP-CCB adverse effects (e.g., edema, hypotension) CYP3A4 Inhibitors may increase serum concentration of DHP-CCB **MOI**: inhibition of CYP3A4	-
Non-DHP-CCB (As CYP3A4 inhibitors)	Diltiazem/Verapamil- Nilotinib (substrate) **Minor DDI**: monitor for increased nilotinib toxicities CYP3A4 Inhibitors may increase serum concentration of nilotinib **MOI**: inhibition of CYP3A4	Diltiazem/Verapamil- Bosutinib (substrate) **Moderate DDI**: avoid combination CYP3A4 Inhibitors may increase serum concentration of bosutinib **MOI**: inhibition of CYP3A4 Diltiazem/Verapamil-Ponatinib (substrate) **Moderate DDI**: monitor for increased ponatinib toxicities CYP3A4 Inhibitors may increase serum concentration of ponatinib **MOI**: inhibition of CYP3A4
Non-DHP-CCB (As substrates)	Imatinib-Diltiazem (minor)/Verapamil (moderate) (substrate) **DDI**: monitor for increased side effects (e.g., hypotension, bradycardia) CYP3A4 Inhibitors may increase serum concentration of non-DHP CCB **MOI**: inhibition of CYP3A4 Nilotinib-Diltiazem (minor)/Verapamil (moderate) (substrate) **DDI**: monitor for increased side effects (e.g., hypotension, bradycardia) CYP3A4 Inhibitors may increase serum concentration of non-DHP CCB	-
	**MOI**: inhibition of CYP3A4	
Potassium-sparing diuretics	Eplerenone **Moderate DDI**: use maximum of 25 mg daily for heart failure and 25 mg twice for hypertension CYP3A4 Inhibitors may increase serum concentration of eplerenone **MOI**: inhibition of CYP3A4	-

ACEI, angiotensin-converting enzyme inhibitors; ARB, angiotensin receptor blockers; ARNI, angiotensin receptor and neprilysin inhibitors; CCB, calcium channel blockers; DHP, dihydropyridine; DDI, drug-drug interaction; MOI, mechanism of interaction; PCSK9i, proprotein convertase subtilisin/kexin type 9; TKI, tyrosine kinase inhibitors.

* No DDI with ezetimibe, PCSK9i, or other unmentioned statins.

$ No DDI with ACEI/ARB/ARNI, other beta-blockers, spironolactone, or thiazide and loop diuretics (27).

### Monitoring and follow-up

High risk patients, based on the selected TKI or present comorbidities, require monitoring and regular follow-up (Figure 1), because risk factors accumulate over time and eventually lead to adverse cardiovascular events (2). A multidisciplinary approach can offer a good opportunity to optimize management. Involvement of a cardio-oncologist or having a cardio-oncology service may offer an individualized approach to ensure TKI therapy benefit, cardiovascular risk prevention, and optimal comorbidity treatment (1, 2).

## Conclusion

Adverse cardiovascular events are considered a major concern of TKI therapy for the treatment of CML. The underlying mechanism is not fully understood but it is related to the inhibition of the non-BCR-ABL kinases. Patients with CML usually have baseline comorbidities which can impact survival. Hypertension, diabetes, and dyslipidemia are among the most common comorbidities. The existing or TKI-associated comorbidities necessitate treatment. The “ABCDE” approach is a concise guide for the primary and secondary prevention of cardiovascular adverse events in the absence of dedicated guidelines. Monitoring and regular follow-up with cardio-oncology service should be an integral part of management.

## Author contributions

All authors listed have made a substantial, direct, and intellectual contribution to the work and approved it for publication.
